# Spatial–Temporal Sensing and Utilization in Full Duplex Spectrum-Heterogeneous Cognitive Radio Networks for the Internet of Things

**DOI:** 10.3390/s19061441

**Published:** 2019-03-23

**Authors:** Waqas Khalid, Heejung Yu

**Affiliations:** Department of Information and Communication Engineering, Yeungnam University, Gyeongsan 38541, Korea; waqas283@gmail.com

**Keywords:** cognitive radio, IoT, full duplex, spectrum sensing, spatial–temporal spectral-holes, utilization of spectrum

## Abstract

The continuous growth of interconnected devices in the Internet of Things (IoT) presents a challenge in terms of network resources. Cognitive radio (CR) is a promising technology that can address the IoT spectral demands by enabling an opportunistic spectrum access (OSA) scheme. The application of full duplex (FD) radios in spectrum sensing enables secondary users (SUs) to perform sensing and transmission simultaneously, and improves the utilization of the spectrum. However, random and dense distributions of FD-enabled SU transmitters (FD-SU TXs) with sensing capabilities in small-cell CR-IoT environments poses new challenges, and creates heterogeneous environments with different spectral opportunities. In this paper, we propose a spatial and temporal spectral-hole sensing framework for FD-SU TXs deployed in CR-IoT spectrum-heterogeneous environment. Incorporating the proposed sensing model, we present the analytical formulation and an evaluation of a utilization of spectrum (UoS) scheme for FD-SU TXs present at different spatial positions. The numerical results are evaluated under different network and sensing parameters to examine the sensitivities of different parameters. It is demonstrated that self-interference, primary user activity level, and the sensing outcomes in spatial and temporal domains have a significant influence on the utilization performance of spectrum.

## 1. Introduction

Recent developments in wireless communication have presented a new networking paradigm, Internet of Things (IoT) [1]. IoT is a network of interconnected devices including sensors, health-care devices, home appliances, autonomous cars, and many others. IoT devices are connected to the internet and are uniquely addressable. IoT promises machine-to-machine (M2M) and human-to-machine (H2M) communications any-time, anyplace, with anyone, using any network (or service) [2]. However, maintaining continuous connectivity and the enormous number of IoT devices present challenges to a radio network. The demands of high bandwidth and spectrum resources for IoT applications lead to the spectrum scarcity [3]. Traditional wireless spectrum standards rely on the static spectrum allocation policies where specific frequency bands are assigned to a specific licensed service and its users. Unlicensed users are not authorized to access the licensed bands, resulting in the underutilized bands. Such policies cause unbalanced utilization of spectrum and degrade the spectral efficiency. Thus, static spectrum allocation policies are insufficient to address the high demands of spectrum resources required for the wireless access of large number of IoT sensor devices [4]. Spectrum allocation flexibility and spectrum utilization efficiency, required for IoT, can be achieved with different proposed technologies such as long term evolution (LTE) wireless local area networks (WLAN) aggregation (LWA), non-orthogonal multiple access (NOMA), operations in millimeter-wave band, LTE over unlicensed band (LTE-U), ultra-dense 5G small cells, and software-defined cognitive radio network (SD-CRN) [5].

### 1.1. Motivations for Using SD-CRNs in IoT

SD-CRN is a state-of-the-art communication paradigm, and is one of the potential technologies adopted for the IoT and other futuristic M2M applications [2,3,4,5,6,7]. SD-CRN exploits the dynamic spectrum allocation (DSA) and spectrum diversity to address the spectrum scarcity and under-utilization issues. Enabling cognitive radio (CR) features in IoT (CR-IoT) helps alleviate congestion in the network; hence, increases the utilization efficiency of spectrum, and helps to meet the spectrum demands of the future IoT. The proposed model is based on the case-study scenarios where IoT devices (with CR features) coexist (share a common frequency band) with any licensed primary cellular network, and can dynamically access the spectrum assigned to a licensed primary network [8,9]. It is not spectral- and cost-efficient for network service providers to utilize an additional licensed spectrum only for IoT services. Therefore, spectrum sharing based on CR features is needed for IoT networks. For (resource/power) constrained IoT devices, simple signal processing techniques are needed to save energy, and reduce system cost. Hence, the simplest non-coherent energy detection (as sensing technique) is preferred as in the previous research [10,11]. In our proposed system, we also consider energy based sensing. Moreover, if IoT devices are restricted to perform the sensing procedure (due to resource/power constraints), the sensing procedure can be employed only for the fusion center (FC) [12]. The SUs in CR-IoT exploit the spectrum under the interference constraint and ensure uninterrupted M2M or human-based LTE communications. The CR features also provide a cognitive facility to the IoT devices that are required to make smart decisions regarding the spectrum and to perform intelligent operations by analyzing the network conditions. The multi-tiered hierarchy in CRNs categorizes users into two types: primary users (PUs) and secondary users (SUs). The DSA techniques enable time division multiple access (TDMA)-based spectrum sharing between the PUs and SUs. However, the spectral efficiency (or spectrum utilization) of CRNs depends significantly on the successful integration of the PUs and SUs. It requires that the SUs be capable of sensing and keeping track of primary transmissions precisely. The three main types of DSA-based approaches are overlay, underlay, and interweave [13,14,15]. In an underlay approach, the SU and PU can transmit simultaneously but with a constraint for the SU to reduce its transmission power when the PU is active. In overlay, an SU transmits simultaneously with a PU, and assists the primary transmissions with certain relaying techniques. In interweave (opportunistic spectrum access (OSA)-based approach), an SU exploits the spectral opportunities and can transmit only when an idle spectral slot is sensed to be idle. In Interweave-based CR systems, the SUs exploit the spectral opportunities through the spectrum sensing. Spectrum sensing is performed either independently or in a cooperative manner. Perfect spectrum sensing is impossible to achieve in the realistic wireless scenarios. Thus, the sensing errors due to imperfect sensing must be considered for the precise analysis of the CR-IoT. The sensing performance can be enhanced by minimizing the sensing errors. Further, the sum rate of the network, as well as the utilization for the PU and SU nodes, can be improved by choosing the optimal sensing operating points under the imperfect sensing scenarios [5,6,7,15,16]. In summary, integrating optimal CR technology into IoT can alleviate the spectrum scarcity issues and contribute in the process of IoT developments.

### 1.2. Half Duplex (HD) and Full Duplex (FD) Radios

Half duplex (HD) [16,17,18], a traditional SD-CRN operation mode, is based on a time-slotted frame structure where the sensing phase is scheduled sequentially with the transmission phase. In HD-CRNs, the node is either in the sensing or transmission mode; thus unable to receive and transmit on the same frequency simultaneously. An increase in the duration of the sensing phase reduces the overall system capacity; an increase in the duration of the transmission phase impairs the sensing accuracy. The other drawbacks of HD-based operations include: (i) sacrifice of duration for the sensing phase in each time slot, (ii) spectrum collision or waste when PUs arrive or leave during the transmission phase, and (iii) requirement for transmission to be divided into small discontinuous slots even if the spectral hole is long and continuous. Such limitations in HD-CRNs result in the inefficient use of system resources. To consider the above limitations, in-band full duplex (FD) radios have been proposed [18,19,20]. The FD-CRNs can simultaneously perform sensing and transmission in each time slot. The FD-enabled nodes are able to use the spectral holes uninterruptedly and continuously. Interference to the primary network is minimized with the continuous sensing in FD-CRNs. Thus, FD-CRNs have considerable potential to enhance the spectrum utilization and overall system capacity. As a downside, FD-CRNs are achieved at a cost of increased energy consumption and hardware complexity. The major limitation of FD radios is the self-interference (SI) of transceivers because of small spacing and power difference between the transmitting and receiving antennas. Recent developments in potential FD techniques, i.e., SI suppression (SIS) and SI cancellation (SIC) have facilitated the implementation of radical FD-CRN systems. However, imperfect SIS and SIC are practical achievable, resulting in non-zero residual SI power. The performance of FD radios highly depends on these SI mitigation capabilities. Excessive SI can even result in the reduced capacity for FD systems, even falling below that of HD systems [20,21,22].

For ease of reference, we summarize our commonly used notations in Table 1.

### 1.3. Main Contributions

This paper extends our research on the utilization of spectrum (UoS) [5,6]. In [5], the proposed study was conducted for the multichannel scenarios considering imperfect sensing and spectrum handoffs. The utilization of spectrum of the SU nodes, opportunistically operating on different PU channels, was investigated under different PU and SU co-existing network topologies. In our previous studies, the UoS was investigated only for the HD radios deployed in time-slotted CRNs. In addition, only a one-dimensional temporal spectral hole-sensing model was considered for the synchronous PU activities. This paper is inspired by the advances in FD techniques, making FD-CRN a potential candidate to complement and sustain the demands of future dense IoT network. The main aspects of the proposed work are as follows:

1. The realistic small-cell CR-IoT scenario is considered where FD-enabled SU transmitters (FD-SU TXs) with sensing capabilities are distributed randomly across the transmission region of primary network. Such a dense distribution of small-cell FD-SU TXs creates spectrum-heterogeneous environment with different spectral opportunities. In this regards, we propose a two-dimensional spatial–temporal spectral hole-sensing model for FD-SU TXs deployed in CR-IoT spectrum-heterogeneous environment, and incorporate the traffic variations of PU both in time and space domains.
2. As a performance metric of the spatial–temporal sensing model, we propose and evaluate the probability of finding the non-availability of spatial–temporal spectral holes, and the probability of missing the availability of spatial–temporal spectral holes.
3. Incorporating the proposed sensing model, we present an analytical formulation and evaluation of a UoS scheme for the FD-SU TXs present at different spatial positions. The performance of UoS scheme is investigated in terms of average number of sensing slots used for the successful secondary communication in each time-slotted frame.

### 1.4. Paper Organization

The remainder of this paper is organized as follows. In Section 2, the system model is described. In Section 3, we describe the proposed spatial–temporal spectral hole-sensing model. Section 4 presents the performance metric for the spatial–temporal spectral hole-sensing model. Section 5 presents the UoS scheme. Section 6 provides the results and discussion. Section 7 concludes the paper, and presents the future work.

## 2. System Model

In this section, we present the system model with a PU spectrum occupancy and frame structure of FD-SU TXs.

### 2.1. PU Spectrum Occupancy Model

**Figure 1:**
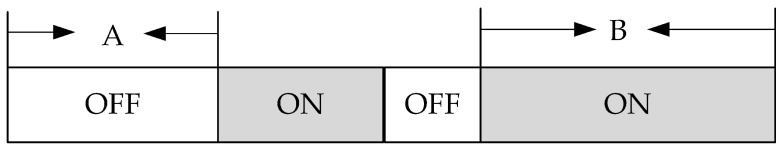
Activity model for primary user (PU).

We assume that the PU is always authorized to use the channel. The PU traffic across the channel is modeled by the discrete-time semi-Markov (two-state) process [22,23,24], as shown in Figure 1. Without loss of generality, it is assumed that the durations of the OFF and ON states are represented by the random variables (RVs) *A* and *B*, respectively. The RVs *A* and *B* follow exponential distribution with mean durations *R* and *S*, respectively. These distributions are to be independent. At any time, the probabilities that the PU is in ON or OFF state are given by $P_{ON}$ = *S*/S+R, and $P_{OFF}$ = *R*/R+S, respectively.

### 2.2. Frame Structure of FD-SU TXs

**Figure 2:**
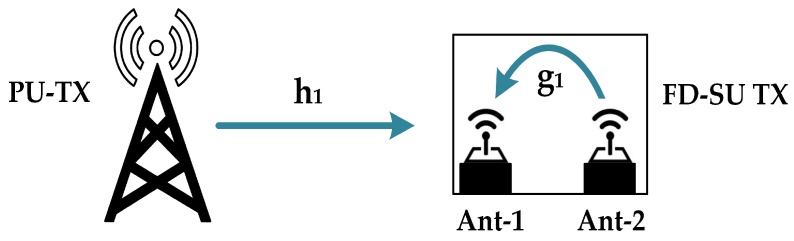
Network model with PU and full duplex-enabled secondary user transmitter (FD-SU TX).

**Figure 3:**
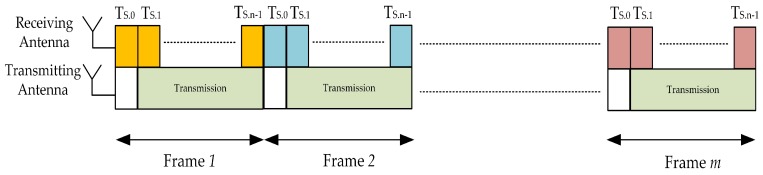
Time-slotted frame structure of FD-SU TXs.

The FD-SU TXs consist of imperfect SIS enabled narrowband antennas, i.e., Ant−1 and Ant−2. As shown in Figure 2, Ant−1 (a receive antenna) performs the sensing procedure to locate the spectral holes and Ant−2 (a transmit antenna) transmits when spectral holes are available [22,23,24,25,26]. In traditional time-slotted FD frame structure, sensing procedure is continuous, i.e., extends to the entire duration. The drawbacks of such frame structure are: (i) change of PU state needs to be consider in the sensing procedure when PU and SU are not synchronized (PU state, in each frame, is not consistent), which normally degrades the detection performance [25], and (ii) time required to make a decision regarding the presence of PU extends to the entire time-slotted frame [26]. The considered time-slotted frame structure for FD-SU TX is shown in Figure 3, in which sensing duration is divided into *n* consecutive short sensing slots (labelled as $T_{S.0}$, $T_{S.1}$,... and $T_{S.n-1}$), and a sensing procedure is performed at each sensing slot. It is assumed that the sensing-slots (in each time-slotted frame) are of same duration. The number of samples in each sensing-slot is *N*, and is expressed as *M*/*n*, where *M* is the number of total samples in each time-slotted frame. The constraint for an asynchronized case is much relax as the duration of each sensing-slot is shorter. Therefore, a consistent PU state, in each sensing-slot, is a more realistic assumption than in the traditional time-slotted FD frame structure, and is considered in the proposed system. The initial (first) sensing-slot in each time-slotted frame is HD (FD-SU TX performs sensing only) to avoid collision at the start. If a PU is not detected at the end of HD sensing slot ($T_{S.0}$ in Figure 3), FD-SU TX initiates its transmission and sensing simultaneously. If a PU is detected at the end of HD sensing slot, FD-SU TX do not transmit and continue to perform the sensing procedure until channel is available in the next time-slotted frame (which also starts with a HD sensing-slot). The reason of continuous sensing is that we do not introduce any switching mechanism for sensing procedure at the receiving antennas of resource constraint IoT devices (FD-SU TXs). Similarly, if a PU is detected at the end of any FD sensing slot, FD-SU TX aborts its transmission until the next time-slotted frame. The motivation behind the considered time-slotted frame structure is to account for the tradeoff between the sensing efficiency and timelines in detecting the PU. An increment in the duration of sensing slots reduces sensing errors; however, it increases the delay of detecting the PU. Conversely, a reduction in the duration of sensing slots reduces the delay of detecting the PU, with the cost of increased sensing errors. The sensed signal during the FD sensing slots ($T_{S.1}$–$T_{S.n-1}$ in Figure 3) is corrupted by a self-interference signal. The formulation of the sensing procedure at FD-SU TX is considered for both types of sensing slots: (i) HD sensing slots (when FD-SU TX is not transmitting) and (ii) FD sensing slots (when FD-SU TX is transmitting). The energy detection (ED) scheme is most commonly used for channel sensing because of its low computational and implementation complexities. Moreover, it does not require prior knowledge of the PU signal parameters [24,25,26,27,28,29,30,31,32]. The secondary network does not guarantee on-time services, i.e., quality of service (QoS) is not guaranteed. Hence, it is assumed that the considered FD-SU TX always has packets to transmit [27].

## 3. Spatial–Temporal Spectral Hole-Sensing Model

**Figure 4:**
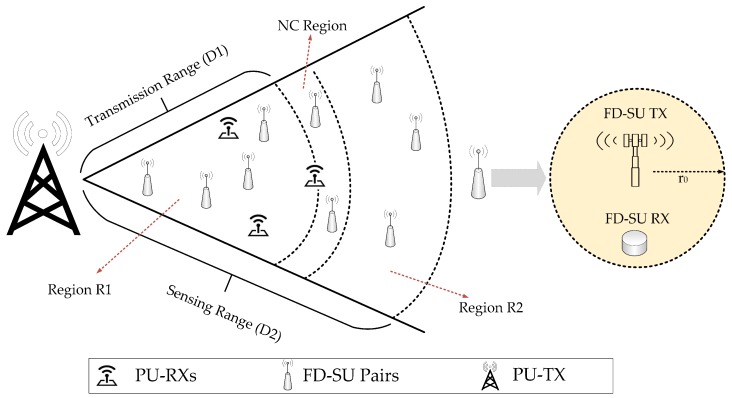
Cognitive radio (CR)-Internet of Things (IoT) spectrum-heterogeneous environment.

In this section, we formulate a spatial–temporal spectral hole-sensing model for FD-SU TXs deployed in CR-IoT spectrum-heterogeneous environment. As shown in Figure 4, we consider a primary network, i.e., a single primary transmitter (PU-TX), multiple primary receivers (PU-RXs), and a secondary network, i.e., *z* number of IoT devices or FD-SU pairs (FD-SU TXs and FD-SU RXs) [5,10,16]. The proposed system can be considered for both downlink and uplink scenarios, i.e., PU-TX as base station (BS) and mobile station (MS). Because there is no difference in the sensing of PU signal at a given time and frequency, the results of the paper are valid for both scenarios. The proposed system depends only on the transmission range of PU-TX, and do not depend on the number of PU-RXs. The performance of channel sensing in temporal domain with multiple PUs has been proposed [33]. However, the authors did not consider the variation of signal power of each PU-TX due to the presence of active surrounding PU-TXs. Given the stochastic nature of PU ON/OFF pattern, the analytical modelling of (two dimensional) spatial-temporal spectral holes sensing framework in the presence of multiple PUs is challenging. To the best of our knowledge, the analysis of spatial–temporal spectral holes with multiple PU-TXs has not been addressed [29,30,31,32]. The spectrum sensing system model [33] with multiple PUs can be extended for the temporal-spatial analysis. However, we consider a single user primary network as the main contribution of the proposed work is to consider the traffic variations of PU in both time and space domains, and characterize and evaluate the sensing and UoS performance of FD-SU pairs present at different spatial positions. The consideration of multiple PUs (cellular users) is added in the future work. In the proposed work, a single PU-TX case is based on the assumption that a single PU-TX has a much higher signal power than any other PU-TXs (in a given frequency and time) in the considered geographical area. Moreover, the transmission range of the primary network is much greater than the secondary IoT network employing low power transmission. In addition, secondary receivers only sense the signal energy, i.e., do not distinguish (decode) the signals from different PU-TXs. Therefore, we can realistically assume that any FD-SU pair is effected by a single PU-TX. We also consider that a single FD-SU pair is assigned to a time-slotted primary channel at a time. Hence, the proposed model do not include the scheduling mechanism between the FD-SU TXs, and only considers the transmission-scheduling of the individual FD-SU pairs in each time slot (illustrated in Figure 3). At a given time and frequency band, we consider only one SU pair by assuming the orthogonal multiple access, e.g., TDMA, FDMA, OFDMA. We considers that all the FD-SU TXs are equipped with the sensing capability. The transmission radius of an FD-SU TX is $r_{0}$. The transmission region of the PU (with radius D1 from PU-TX) is R1, and is determined by the receiving sensitivity of PU [29,30]. If a primary receiver (PU-RX) is outside R1, it will not work properly. The FD-SU pairs are randomly distributed in an area (with radius D2 from PU-TX), which is closely related to the sensing sensitivity of FD-SU TXs. Any FD-SU TX, outside this area, will not be able to detect the primary signal. The R2 is the region outside of R1. We considers a typical scenario (i.e., D2>D1), in which FD-SU TXs have a higher sensing sensitivity than the receiver sensitivity of PU-RXs (to avoid interference with the primary transmission) [29,30,31,32,33]. Interference to the PU-RX is not permitted. Hence, FD-SU TXs in R1 have the temporal spectral opportunities only, and the traffic variations of PU are considered in the time domain. The FD-SU TXs in R2 have the spatial spectral opportunities (can access the channel anytime). However, FD-SU TXs lose their spatial spectral opportunities when detect a PU-TX. The FD-SU TXs in R2, near to the PU-RXs, can cause harmful interference and are referred to be present in the NC Region. The NC Region is determined by the interference constraint from the PU-RXs, and the peak transmission power of the FD-SU TXs [29]. An appropriate power control, which is beyond the scope of this paper, is an essential requirement to allow these FD-SU TXs to exploit their spectral opportunities. Hence, FD-SU TXs in the NC Region are not considered in the proposed work.

In the considered network, there are two potential signal sources, i.e., PU-TX and FD-SU TX. Thus, we define four tests of the hypothesis as follows,

- $H_{00}$ PU-TX is OFF and FD-SU TX is not transmitting.
- $H_{01}$ PU-TX is ON and FD-SU TX is not transmitting.
- $H_{10}$ PU-TX is OFF and FD-SU TX is transmitting.
- $H_{11}$ PU-TX is ON and FD-SU TX is transmitting.

The traditional temporal spectral-holes during HD and FD sensing slots are respectively expressed as following hypothesis testing problems,

(1) $$S\left(i\right)=\left\{\begin{array}{cc} n(i), & H_{00}\\ h_{1}s_{1}\left(i\right)+n\left(i\right), & H_{01} \end{array}\right.$$

(2) $$S\left(i\right)=\left\{\begin{array}{cc} g_{1}s_{2}\left(i\right)+n\left(i\right), & H_{10}\\ h_{1}s_{1}\left(i\right)+g_{1}s_{2}\left(i\right)+n\left(i\right), & H_{11} \end{array}\right.$$

Here, n(i) denotes the *i*-th noise signal sample at FD-SU TX, and is considered to be complex Gaussian with zero mean and variance $\sigma_{n}^{2}$. $s_{1}\left(i\right)$ is the *i*th sample of the transmitted signal of PU-TX, and is assumed to be phase-shift keying (PSK)-modulated with variance $\sigma_{s_{1}}^{2}$ [34,35]. $s_{2}\left(i\right)$ is the *i*-th sample of the transmitted signal of FD-SU TX (before SIS) and is also assumed to be PSK-modulated with variance $\sigma_{s_{2}}^{2}$. $h_{1}$ is the complex Gaussian channel coefficient between PU-TX and FD-SU TX with zero mean and variance $\sigma_{h_{1}}^{2}$. $g_{1}$ represents the self-interference channel between the Ant−1 and Ant−2 of FD-SU TX, which follows the complex Gaussian random variable with zero mean and variance $\sigma_{g_{1}}^{2}$. In (1) and (2), *i* = 0, 1… *N* – 1 is the sample index.

In general, the traditional spatial spectral holes for SU node can be expressed as the following hypothesis-testing problem [29,30,31,32],

(3) $$\left\{\begin{array}{cc} S_{A}:\;\; & SU\;node\;is\;present\;in\;R2\\ S_{B}:\;\; & SU\;node\;is\;present\;in\;R1, \end{array}\right.$$

where hypothesis $S_{A}$ denotes the availability of spatial spectral holes, and hypothesis $S_{B}$ denotes the non-availability of spatial spectral holes.

From a joint (two-dimensional) sensing perspective, spatial–temporal spectral holes for SU node can be described as the following hypothesis testing problem [29,30,31,32],

(4) $$\left\{\begin{array}{cc} O_{A}:\;\; & H_{0}\cup S_{A}\\ O_{B}:\;\; & H_{1}\cap S_{B}, \end{array}\right.$$

where $O_{A}$ denotes the case where spatial–temporal spectral holes are available, either because of the absence of PU ($H_{0}$), or the PU is present and the SU node locates in R2 ($S_{A}$). Similarly, $O_{B}$ denotes the case that spatial–temporal spectral holes are not available because of the presence of PU ($H_{1}$) and the SU node locates in R1 ($S_{B}$).

Considering Equations (1), (2), and (4), we model the availability of spatial–temporal spectral holes for FD-SU TX as the following composite hypothesis testing problems

(5) $$O_{A}^{0}:S\left(i\right)=\left\{\begin{array}{cc} n(i) & \left\{H_{00}\right\}\\ h_{1}s_{1}\left(i\right)+n\left(i\right) & \left\{H_{01}\right\},\;\;S_{A}, \end{array}\right.$$

(6) $$O_{A}^{1}:S\left(i\right)=\left\{\begin{array}{cc} g_{1}s_{2}\left(i\right)+n\left(i\right) & \left\{H_{10}\right\}\\ h_{1}s_{1}\left(i\right)+g_{1}s_{2}\left(i\right)+n\left(i\right) & \left\{H_{11}\right\},\;\;S_{A}, \end{array}\right.$$

where $O_{A}^{0}$ denotes the availability of spatial–temporal spectral holes during HD sensing slots either because of $H_{00}$, or $H_{01}$ and FD-SU TX is present in R2 ($S_{A}$). Similarly, $O_{A}^{1}$ denotes the availability of spatial–temporal spectral holes during FD sensing slots either because of $H_{10}$, or $H_{11}$ and FD-SU TX is present in R2 ($S_{A}$).

Considering Equations (1), (2), and (4), we now model the non-availability of spatial–temporal spectral holes for FD-SU TX as the following composite hypothesis testing problems

(7) $$O_{B}^{0}:S\left(i\right)=\left\{\begin{array}{cc} h_{1}s_{1}\left(i\right)+n\left(i\right) & \left\{H_{01}\right\},\;\;S_{B}, \end{array}\right.$$

(8) $$O_{B}^{1}:S\left(i\right)=\left\{\begin{array}{cc} h_{1}s_{1}\left(i\right)+g_{1}s_{2}\left(i\right)+n\left(i\right) & \left\{H_{11}\right\},\;\;S_{B}, \end{array}\right.$$

where $O_{B}^{0}$ denotes the non-availability of spatial–temporal spectral holes during HD sensing slots because of $H_{01}$ and FD-SU TX is present in R1 ($S_{B}$). Similarly, $O_{B}^{1}$ denotes the non-availability of spatial–temporal spectral holes during FD sensing slots because of $H_{11}$ and FD-SU TX is present in R1 ($S_{B}$).

It can be observed that the availability/non-availability of spatial–temporal spectral holes (during HD and FD sensing slots) in Equations (5)–(8) is a combination of availability/non-availability of pure temporal and spatial spectral holes in Equations (1)–(4). However, spatial–temporal spectral hole-sensing model incorporates the traffic variations of the PU in both domains (time and space) for the FD-SU TXs present at different spatial positions.

## 4. Performance Metric for Spatial–Temporal Spectral Hole-Sensing Model

In this section, we introduce the performance metric to guide the spatial–temporal spectral hole-sensing model. Closed-form expressions of sensing probabilities are proposed. The sensing probabilities are: (i) the probability of finding the non-availability of spatial–temporal spectral holes and (ii) the probability of missing the availability of spatial–temporal spectral holes.

The probabilities of finding the non-availability of spatial–temporal spectral holes during HD and FD sensing slots are respectively expressed as

(9) $$P_{F.nST}^{0}\overset{\Delta }{=}Pr\left\{\Psi_{ST}^{0}=O_{B}^{0}\;|\;O_{B}^{0}\right\}$$

(10) $$P_{F.nST}^{1}\overset{\Delta }{=}Pr\left\{\Psi_{ST}^{1}=O_{B}^{1}\;|\;O_{B}^{1}\right\},$$

where $\Psi_{ST}^{0}$ and $\Psi_{ST}^{1}$ denote the decision functions for spatial–temporal spectral holes during HD and FD sensing-slots, respectively.

Based on the detail derivation in [29] and extending it separately for HD and FD sensing slots, the probabilities of finding the non-availability of spatial–temporal spectral holes are expressed by the temporal states of the PU and the spatial positions of FD-SU TX as

(11) $$P_{F.nST}^{0}=Pr\left\{T\left(S\right)>\phi_{1}\;|\;H_{01}\right\},\;\;S_{B}$$

(12) $$P_{F.nST}^{1}=Pr\left\{T\left(S\right)>\phi_{2}\;|\;H_{11}\right\},\;\;S_{B},$$

where $P_{F.nST}^{0}$ and $P_{F.nST}^{1}$ denote the probabilities of finding the non-availability of spatial–temporal spectral holes during HD and FD sensing-slots, respectively. $\phi_{1}$ and $\phi_{2}$ are the detection thresholds during HD and FD sensing slots, respectively. T(S) denotes the test statistics, and is expressed as $\frac{1}{N}\sum_{i=0}^{N-1}{\left|S(i)\right|}^{2}$.

Similarly, the probabilities of missing the availability of spatial–temporal spectral holes during HD and FD sensing slots are respectively expressed as

(13) $$P_{Mi.ST}^{0}\overset{\Delta }{=}Pr\left\{\Psi_{ST}^{0}=O_{B}^{0}\;|\;O_{A}^{0}\right\}$$

(14) $$P_{Mi.ST}^{1}\overset{\Delta }{=}Pr\left\{\Psi_{ST}^{1}=O_{B}^{1}\;|\;O_{A}^{1}\right\}.$$

In a similar fashion, extending the derivation in [29] separately for HD and FD sensing slots, the probabilities of missing the availability of spatial–temporal spectral holes are also expressed by the temporal states of the PU and spatial positions of the FD-SU TX as

(15) $$P_{Mi.ST}^{0}=\left\{\begin{array}{cc} Pr\left\{T\left(S\right)>\phi_{1}\;|\;H_{00}\right\}P_{OFF}, & S_{B}\\ Pr\left\{T\left(S\right)>\phi_{1}\;|\;H_{00}\right\}P_{OFF}+Pr\left\{T\left(S\right)>\phi_{1}\;|\;H_{01}\right\}P_{ON}, & S_{A} \end{array}\right.$$

(16) $$P_{Mi.ST}^{1}=\left\{\begin{array}{cc} Pr\left\{T\left(S\right)>\phi_{2}\;|\;H_{10}\right\}P_{OFF}, & S_{B}\\ Pr\left\{T\left(S\right)>\phi_{2}\;|\;H_{10}\right\}P_{OFF}+Pr\left\{T\left(S\right)>\phi_{2}\;|\;H_{11}\right\}P_{ON}, & S_{A}. \end{array}\right.$$

It can be observed that the probabilities of finding the non-availability of spatial–temporal spectral holes ($P_{F.nST}^{0}$, and $P_{F.nST}^{1}$) are related to the FD-SU TXs in R1 only, because the FD-SU TXs in R2 have spatial spectral holes, i.e., can access the channel anytime. The probabilities of missing the availability of spatial–temporal spectral holes ($P_{Mi.ST}^{0}$, and $P_{Mi.ST}^{1}$) for the FD-SU TXs in R1 are determined by the OFF state of the PU, and are represented with an event that the FD-SU TX detects the presence of the PU when the PU is in OFF state, i.e., temporal false alarms. Furthermore, $P_{Mi.ST}^{0}$, and $P_{Mi.ST}^{1}$ for the FD-SU TXs in R2 are determined by each state of the PU, i.e., ON, and OFF states, and are represented with the following two events; (i) FD-SU TX determines the presence of PU when PU is in the OFF state, i.e., temporal false alarms, and (ii) FD-SU TX determines the presence of the PU when the PU is in the ON state, i.e., spatial false alarms.

Given S(i) in Equation (1), and Equation (2) as i.i.d., the mean and variance of T(S) can be expressed as,

(17) $$E\left[T(S)\right]=E\left[{\left|S(i)\right|}^{2}\right],\;\;\;E\left[T(S)\right]=\frac{1}{N}var\left[{\left|S(i)\right|}^{2}\right].$$

Using the central limit theorem (CLT), we obtain the distribution of T(S) given each of the defined hypotheses for HD and FD sensing slots. For a large *N*, the probability density functions (pdf) of T(S) given $H_{00}$, $H_{10}$, $H_{01}$, and $H_{11}$ are complex-valued Gaussian with the following mean and variance,

(18) $$E\left[T\left(S\right)|H_{00}\right]=\sigma_{n}^{2},\;\;\;var\left[T\left(S\right)|H_{00}\right]=\frac{1}{N}\left[\sigma_{n}^{4}\right]$$

(19) $$E\left[T\left(S\right)|H_{10}\right]=\sigma_{n}^{2}\left(\gamma_{1}+1\right),\;\;\;var\left[T\left(S\right)|H_{10}\right]=\frac{1}{N}\left[\sigma_{n}^{4}{\left(\gamma_{1}+1\right)}^{2}\right]$$

(20) $$E\left[T\left(S\right)|H_{01}\right]=\sigma_{n}^{2}\left(\gamma_{p}+1\right),\;\;\;var\left[T\left(S\right)|H_{01}\right]=\frac{1}{N}\left[\sigma_{n}^{4}{\left(\gamma_{p}+1\right)}^{2}\right]$$

(21) $$E\left[T\left(S\right)|H_{11}\right]=\sigma_{n}^{2}\left(\gamma_{1}+\gamma_{p}+1\right),\;\;\;var\left[T\left(S\right)|H_{01}\right]=\frac{1}{N}\left[\sigma_{n}^{4}{\left(\gamma_{1}+\gamma_{p}+1\right)}^{2}\right],$$

where $\gamma_{p}$ is the instantaneous sensing SNR at the FD-SU TX, and is expressed as $\frac{\sigma_{h_{1}}^{2}\sigma_{s_{1}}^{2}}{\sigma_{n}^{2}}$. Tha parameter $\gamma_{1}$ is the INR at the FD-SU TX due to the SU transmission, and is expressed as $\frac{X^{2}\sigma_{s_{2}}^{2}}{\sigma_{n}^{2}}$. *X* is an SIS factor that quantifies the degree of imperfect SIS, $X\in \left[0,1\right]$. *X* represents the ratio between the residual self-interference and the transmitting power of FD-SU TX [28]. If X=0 (perfect SIS), FD-SU TX can completely suppress its residual self-interference. Substituting the statistical properties under each hypothesis (18)–(21), we obtain,

(22) $$Pr\left\{T\left(S\right)>\phi_{1}\;|\;H_{00}\right\}=Q\left[\left(\frac{\phi_{1}}{\sigma_{n}^{2}}-1\right)\sqrt{N}\right]$$

(23) $$Pr\left\{T\left(S\right)>\phi_{1}\;|\;H_{01}\right\}=Q\left[\left(\frac{\phi_{1}}{\left(1+\gamma_{p}\right)\sigma_{n}^{2}}-1\right)\sqrt{N}\right]$$

(24) $$Pr\left\{T\left(S\right)>\phi_{2}\;|\;H_{10}\right\}=Q\left[\left(\frac{\phi_{2}}{\left(1+\gamma_{1}\right)\sigma_{n}^{2}}-1\right)\sqrt{N}\right]$$

(25) $$Pr\left\{T\left(S\right)>\phi_{2}\;|\;H_{11}\right\}=Q\left[\left(\frac{\phi_{2}}{\left(1+\gamma_{1}+\gamma_{p}\right)\sigma_{n}^{2}}-1\right)\sqrt{N}\right].$$

The sensing performance during HD and FD sensing slots can be formulated either under the QoS constraint for the PU, i.e., guaranteed protection level to the PU, or the QoS constraint for the FD-SU TX, i.e., guaranteed usability rate of spatial–temporal spectral holes [5]. In the proposed work, we consider the QoS constraint for the PU, i.e., the probability of finding the non-availability of spatial–temporal spectral holes is fixed to the desired value ($\bar{P_{F.nST}^{0}}$, and $\bar{P_{F.nST}^{1}}$), and the corresponding probability of missing the availability of spatial–temporal spectral holes ($P_{Mi.ST}^{0}$, and $P_{Mi.ST}^{1}$), given the sensing parameters, is obtained. Under the considered constraints, ($P_{Mi.ST}^{0}$, and $P_{Mi.ST}^{1}$) for the FD-SU TXs in R1 and R2 can be expressed as 1.8

(26) $$P_{Mi.ST}^{0}=\left\{\begin{array}{cc} Q\left[\frac{\sqrt{N{\left(\gamma_{p}+1\right)}^{2}}Q^{-1}\left(\bar{P_{F.nST}^{0}}\right)+\left(N\gamma_{p}\right)}{\sqrt{N}}\right]P_{OFF}, & S_{B}\\ Q\left[\frac{\sqrt{N{\left(\gamma_{p}+1\right)}^{2}}Q^{-1}\left(\bar{P_{F.nST}^{0}}\right)+\left(N\gamma_{p}\right)}{\sqrt{N}}\right]P_{OFF}+\bar{P_{F.nST}^{0}}P_{ON}, & S_{A} \end{array}\right.$$

(27) $$P_{Mi.ST}^{1}=\left\{\begin{array}{cc} Q\left[\frac{\sqrt{N{\left(\gamma_{1}+\gamma_{p}+1\right)}^{2}}Q^{-1}\left(\bar{P_{F.nST}^{1}}\right)+\left(N\gamma_{p}\right)}{\sqrt{N}\left(\gamma_{1}+1\right)}\right]P_{OFF}, & S_{B}\\ Q\left[\frac{\sqrt{N{\left(\gamma_{1}+\gamma_{p}+1\right)}^{2}}Q^{-1}\left(\bar{P_{F.nST}^{1}}\right)+\left(N\gamma_{p}\right)}{\sqrt{N}\left(\gamma_{1}+1\right)}\right]P_{OFF}+\bar{P_{F.nST}^{1}}P_{ON}, & S_{A}, \end{array}\right.$$

where Q(.) and $Q^{-1}\left(.\right)$ denote the complementary distribution function of the standard Gaussian and its inverse, respectively.

## 5. Utilization of Spectrum (UoS) Scheme

**Figure 5:**
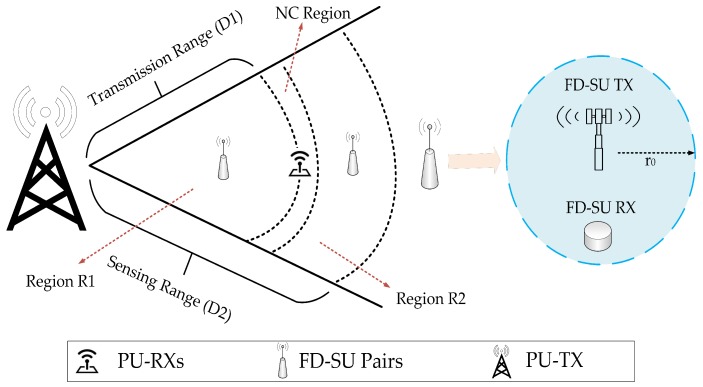
Considered cases of FD-SU TXs.

Based on the proposed spatial–temporal spectral hole-sensing model in Section 3, we present an analytical formulation and evaluation of the UoS scheme for the FD-SU TXs deployed at different spatial positions in CR-IoT spectrum-heterogeneous environment. The UoS performance was evaluated by determining the average number of sensing slots, (τ), used for the successful secondary communication in each time-slotted frame. Because a dense small-cell scenario is considered, i.e., $r_{0}<<D1$, the UoS performance was evaluated for two possible cases, as shown in Figure 5. The cases are: (i) the FD-SU TX is present in R1 (FD-SU pair is completely inside the transmission range of PU) and (ii) the FD-SU TX is present in R2 (FD-SU pair is completely outside the transmission range of PU). As explained earlier, the first sensing slot in each time-slotted frame is always HD, i.e., FD-SU TX only performs the sensing procedure. If the PU is not detected at the end of HD sensing slot, the FD-SU TX initiates its transmission and sensing simultaneously. If the PU is detected at the end of HD (or any FD) sensing slot, the FD-SU TX do not transmit (or stops transmission) until the next cycle (time-slotted frame).

The probability of detecting the spatial–temporal spectral holes during HD and FD sensing slots depends on the sensing outcome, the PU activity, and the spatial position of FD-SU TX. The probability of detecting the spatial–temporal spectral holes in only first *k* number of sensing slots in each time-slotted frame can be defined as

(28) $$P_{ST-h}\left(k\right)=P_{A}\left(k\right)-P_{B}\left(k+1\right).$$

Here, $P_{A}\left(k\right)$ refers to the possible scenarios where spatial–temporal spectral holes are detected in greater or equal to *k* number of sensing slots. Similarly, $P_{B}\left(k+1\right)$ refers to the possible scenarios where spatial–temporal spectral holes are detected in greater or equal to k+1 number of sensing slots. Hence, $P_{A}\left(k\right)$ can be expressed as

(29) $$P_{A}\left(k\right)=\left(1-P_{Mi.ST}^{0}\right)\sum_{j=1}^{k}{\left(1-P_{Mi.ST}^{1}\right)}^{j}.$$

The probabilities of missing the availability of spatial–temporal spectral holes ($P_{Mi.ST}^{0}$, and $P_{Mi.ST}^{1}$) for FD-SU TX in R2 are determined by each state of the PU, and involve both temporal and spatial (excessive) false alarms. Conversely, $P_{Mi.ST}^{0}$ and $P_{Mi.ST}^{1}$ for FD-SU TX in R1 involve only temporal false alarms. It is assumed that all FD-SU TXs are synchronous to their corresponding receivers, and initiates transmission and reception at the same time.

A channel error occurs when the received SNR, because of path loss or deep fading, falls below the considered threshold [5,13]. We consider the same bit error rate (BER) in each time-slotted frame. Hence, the number of transmitted bits is adjusted as per the *k* number of sensing slots in each time-slotted frame. The probability of obtaining an errored secondary packet, owing to channel errors, can be expressed as [5]

(30) $$P_{E}=1-\left[{\left(1-BER\left(\gamma_{s}\right)\right)}^{kPS}\right],$$

where kPS is the packet size, i.e., transmitted number of bits in *k* number of sensing-slots, $\gamma_{s}$ is the SNR of the secondary transmission, and BER is the bit error rate for the considered secondary channel, and can be expressed as [5]

(31) $$BER\left(\gamma_{s}\right)=\frac{1}{4\gamma_{s}}.$$

In our work, we assume that the UoS is contributed to only during the sensing slots during which FD-SU TXs detect the state of the considered channel correctly. Moreover, the access contention for the FD-SU TXs is also not considered.

From Equations (28)–(30), the average number of sensing slots used for a successful secondary communication in each time-slotted frame can be defined as

(32) $$\tau =\sum_{k=1}^{\infty }kP_{ST-h}\left(k\right)\left(1-P_{E}\left(k\right)\right)$$

## 6. Results and Discussion

### 6.1. Simulation Setup

In this section, numerical results are provided to evaluate the performance of proposed spatial–temporal spectral hole-sensing model and UoS scheme for FD-SU TXs with different temporal states and spatial positions. In our simulation setup, the transmission range of the PU node and sensing range of the FD-SU TXs were varied to allow different values of probabilities and sensing parameters. To obtain the numerical results, the key parameters were chosen as follows: kPS = 1000 bits for k=1 and BER = 0.00025. The power of the primary and secondary transmissions was set to be 20 dB and 10 dB, respectively. The variance of the sensing channel was set to $\sigma_{h_{1}}^{2}$ = 0.001, and hence, the instantaneous sensing SNR was −10 dB. The values of the SIS factor (*X*) were considered to be in the range 0.001–0.3. To provide the desired protection level to the PU, the probabilities of finding the non-availability of spatial–temporal spectral holes during HD and FD sensing slots ($\bar{P_{F.nST}^{0}}$, and $\bar{P_{F.nST}^{1}}$) were considered to be in the range 0.7–0.9. Unless otherwise stated, a fair model of PU the status, i.e., $P_{ON}$ = $P_{OFF}$ = 0.5 was considered. The values of these parameters were set accordingly to validate the channels characteristics and network behavior.

### 6.2. Simulation Results

**Figure 6:**
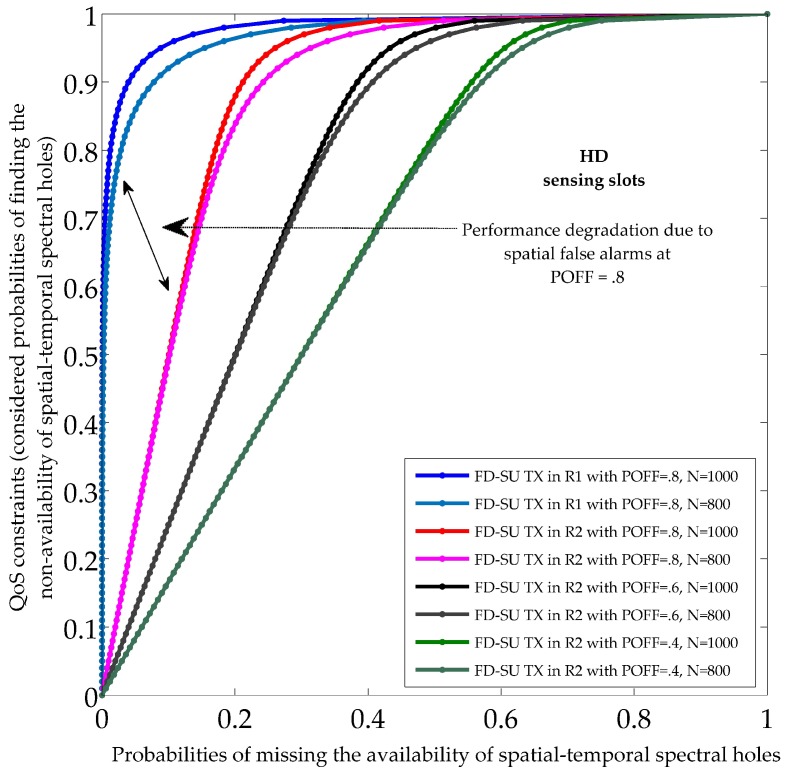
Sensing performance of spatial–temporal spectral holes during the half duplex (HD) sensing slots.

**Figure 7:**
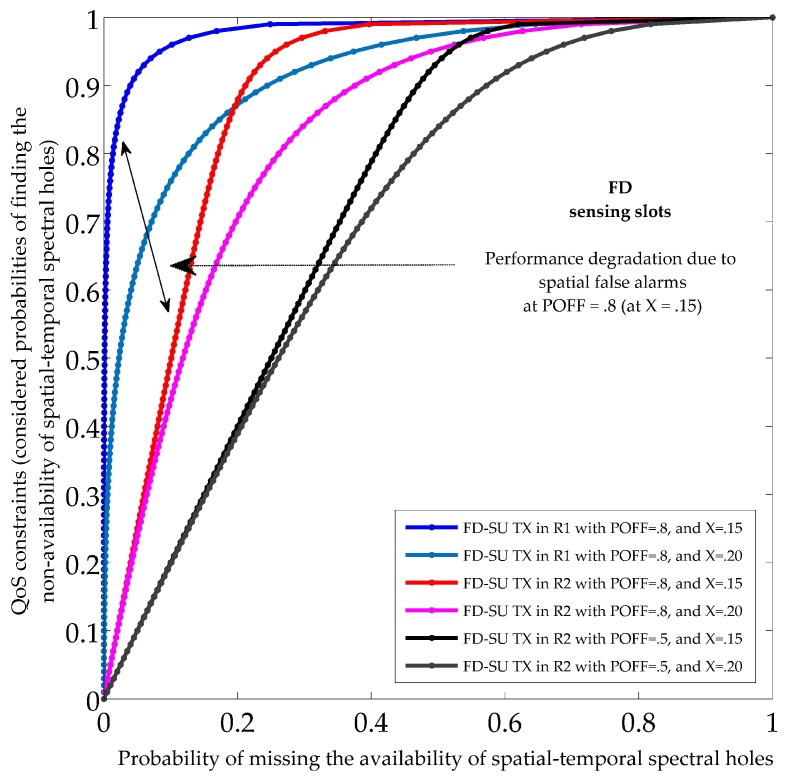
Sensing performance of spatial–temporal spectral holes during the full duplex (FD) sensing slots.

Figure 6 and Figure 7 show the sensing performance during HD and FD sensing slots, respectively, in term of the probabilities of finding the non-availability of spatial–temporal spectral holes, and the probabilities of missing the availability of spatial–temporal spectral holes. There was no self-interference signal during HD sensing slots. However, the sensed signal during FD sensing slots was corrupted with the self-interference signal as FD-SU TX was transmitting simultaneously. The results indicate the existence of different spatial and temporal spectral opportunities for FD-SU TXs present at different spatial positions in CR-IoT spectrum-heterogeneous environment. It can be observed that the probabilities of missing the availability of spatial–temporal spectral holes during HD and FD sensing slots ($P_{Mi.ST}^{0}$, and $P_{Mi.ST}^{1}$) was always greater for FD-SU TXs in R2 than those in R1. This is because the $P_{Mi.ST}^{0}$, and $P_{Mi.ST}^{1}$ for FD-SU TX in R2 involves spatial (excessive) false alarms, determined by ON state of the PU. In detail, temporal false alarms for FD-SU TXs (R1 and R2) arose because of the miss-detection of the absence of a PU. The spatial false alarms for FD-SU TXs in R2 arose because of the detection of presence of a PU despite the fact that the FD-SU TX is outside the transmission region. The spatial and temporal false alarms depend on the usage of channel by the PU, and number of sensing samples, respectively. It can be observed that the reduced use of channel by the PU and a greater number of sensing samples result in fewer spatial and temporal false alarms, respectively, and results in the improved spatial–temporal spectral hole-sensing performance. The influence of different INRs in terms of SIS factors (*X*) over the spatial–temporal spectral hole-sensing performance is shown in Figure 7. The temporal false alarms increased with *X*, which is intuitive because *X* increases the interference power. Thus, our results illustrate the requirement for identifying the optimal range of the secondary transmit power to obtain the desired spatial–temporal spectral hole-sensing performance.

**Figure 8:**
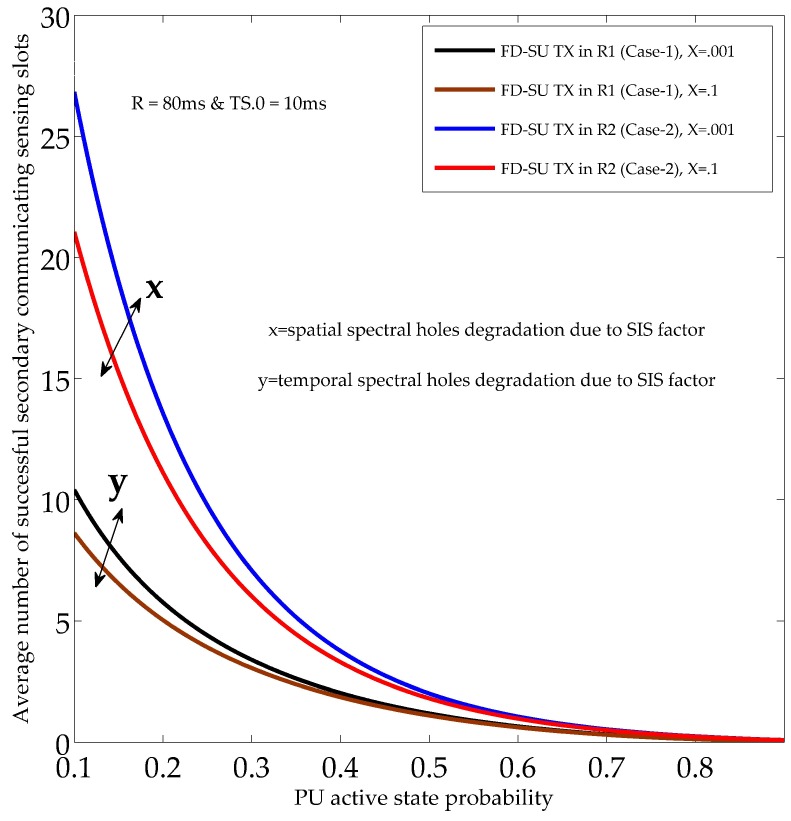
Average number of secondary communicating sensing slots with different PU active state probabilities and SIS factors.

**Figure 9:**
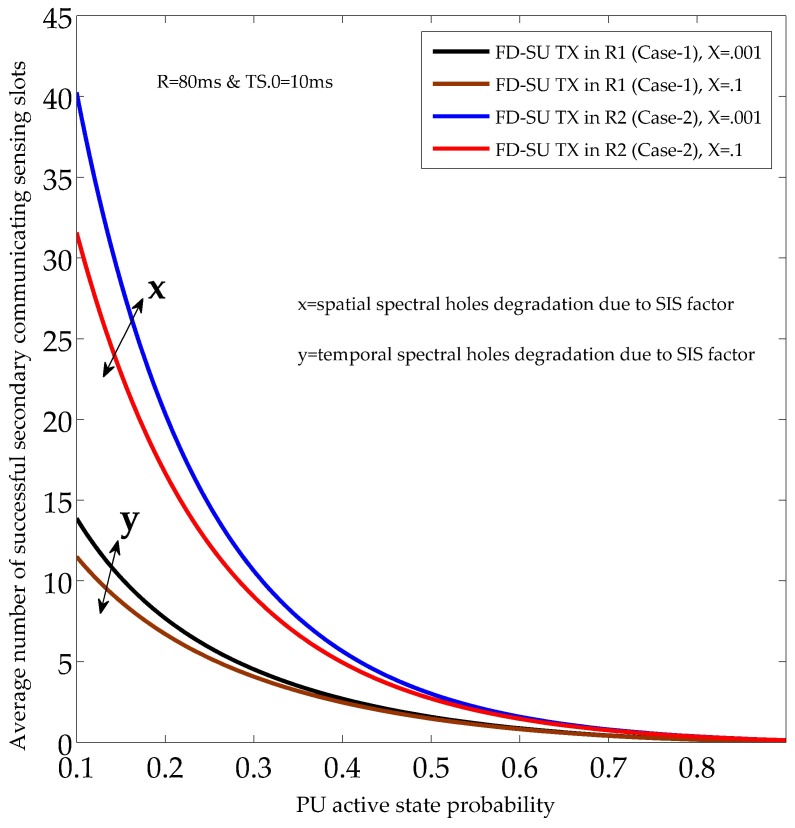
Average number of secondary communicating sensing slots with different PU active state probabilities and SIS factors (at different quality of service (QoS) constraint).

Figure 8 and Figure 9 illustrate the performance of the UoS scheme, and show the average number of sensing slots used for the successful secondary communication (in each time-slotted frame). The performance of the UoS scheme was investigated for different PU active state probabilities ($P_{ON}$), SIS factors, and QoS constraints ($\bar{P_{F.nST}^{0}}$, and $\bar{P_{F.nST}^{1}}$). In Figure 8 and Figure 9, the QoS constraints (during HD and FD sensing-slots) were set to 0.9, and 0.7, respectively. Reduced QoS constraints means less protection to the PU and more spectral opportunities for FD-SU TXs (R1 and R2), and hence, average number of sensing slots used for the successful secondary communication increases. In detail, the results show the reduction in the average number of successful secondary communicating sensing slots with the usage of channel by the PU node, considering a fixed PU mean OFF duration (*R*). The reason is that the spectral opportunities (spatial and temporal) decrease under the considered scenario. The average number of successful secondary communicating sensing slots also reduced with the SIS factors owing to the increase in temporal and spatial false alarms. The results validate the existence of different spectral opportunities in CR-IoT spectrum-heterogeneous environment by demonstrating different average number of communicating sensing slots for R1 and R2. Importantly, the average number of successful secondary communicating sensing slots for FD-SU TX in R1 was less than that of R2. This is because FD-SU TX in R2 can continue to avail the spatial spectral opportunities even when PU is in ON state, which is not the case for FD-SU TX in R1. Hence, our results validate the requirements for the optimal performance of the UoS in CR-IoT spectrum-heterogeneous environment.

**Figure 10:**
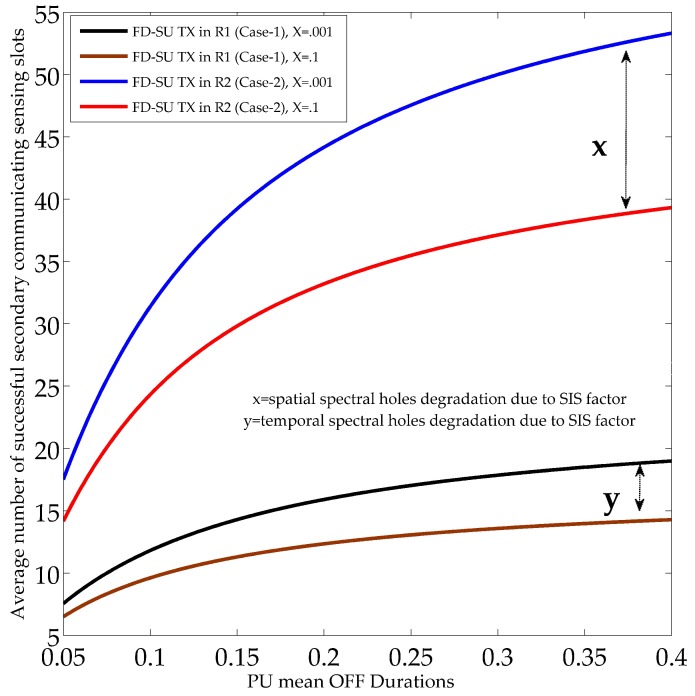
Average number of secondary communicating sensing slots with different PU mean OFF durations and SIS factors.

In Figure 10, the performance of the UoS scheme, in terms of average number of sensing slots used for the successful secondary communication (in each time-slotted frame), is investigated under different PU mean inactive durations (*R*) and SIS factors. The QoS constraints for the probability of finding the non-availability of spatial–temporal spectral holes (during HD and FD sensing-slots) were set to 0.9. The PU active state probability was considered to be 0.1. The results again demonstrate the existence of different spectral opportunities in CR-IoT spectrum-heterogeneous environment, and validate the average number of successful secondary communicating sensing slots for temporal and spatial spectral holes. Importantly, it can be observed that the average number of sensing slots used for the successful secondary communication for all FD-SU TXs (R1 and R2) increased as *R* increased. The reason is that the increase in *R* at fixed PU states probabilities ($P_{ON}$ and $P_{OFF}$) and duration of sensing slots means more sensing slots with temporal and spatial spectral holes. Hence, the average number of successful secondary communicating sensing slots increased. Our results help to identify the ranges of PU mean durations and SIS factors, subject to the spatial positions of FD-SU TXs, for the required sensing performance in spatial and temporal domains.

## 7. Conclusions

CRNs are designed to manage radio resources efficiently by utilizing the spectral holes in licensed frequency bands. The in-band FD technology, a new paradigm for CRNs, allows SUs to simultaneously sense and access the spectral holes. However, the efficient sharing of spectrum between the dynamic PU and FD-enabled SU transmitters (FD-SU TXs), in dense small-cell IoT scenarios, presents challenges that must be addressed. The random and dense distribution of FD-SU TXs with sensing capabilities creates heterogeneous environments with temporal and spatial spectral opportunities. In this regard, we considered the traffic variations of the PU both in time and space domains, and proposed a two-dimensional spatial and temporal spectral hole-sensing model for the FD-SU TXs deployed in CR-IoT spectrum-heterogeneous environment. Incorporating the proposed sensing model, an analytical formulation and evaluation of the UoS scheme was proposed for different FD-SU TXs. The performance of the UoS scheme was investigated in terms of average number of sensing slots used for the successful secondary communication in each time-slotted frame. The numerical results validated the influence of different network and sensing parameters over the proposed spatial–temporal spectral hole-sensing model and UoS scheme. In the future work, we can extend the proposed approach by considering the temporal and spatial variations of idle channels in more complicated IoT-CRN scenarios such as full duplex (cooperative) or ARQ/HARQ enabled SU nodes co-exist with the multiple PU-TXs.

## Figures and Tables

**Table 1:** Summary of adopted notations.

Symbol	Description
$P_{OFF}$	Probability that the PU is in OFF state
$P_{ON}$	Probability that the PU is in ON state
*R*	Mean of OFF durations
*S*	Mean of ON durations
*A*	Random variable for OFF duration
*B*	Random variable for ON duration
*T*	Time-slotted frame length
*N*	Number of sensing samples in each sensing slot
R1	Transmission region
R2	Sensing region
D1	Radius of transmission region
D2	Radius of sensing region
*X*	SIS factor
$\gamma_{p}$	Instantaneous sensing signal-to-noise ratio (SNR)
$\gamma_{1}$	Instantaneous interference-to-noise ratio (INR)
$\gamma_{s}$	SNR of secondary transmissions
